# Groundwater divide shifting due to pumping in a sector of the Doñana aquifer system (SW Spain): environmental implications

**DOI:** 10.1007/s10661-025-13965-z

**Published:** 2025-04-09

**Authors:** Manuel Olías, M. Dolores Basallote, Carlos R. Cánovas, Cristina Pérez-Carral

**Affiliations:** 1 Department of Earth Sciences & Research Center on Natural Resources, Health and the Environment, University of Huelva, Campus “El Carmen”, 21071 Huelva, Spain; 2 Department of Ecology and Coastal Management, Institute of Marine Sciences of Andalusia, CSIC, 11510 Puerto Real, Cádiz, Spain; 3 Department of Agroforestry, University of Huelva, Campus “El Carmen”, 21071 Huelva, Spain

**Keywords:** Shared aquifer, Groundwater overexploitation, Impact on wetlands, Surface and groundwater divide

## Abstract

The Doñana Natural Space (SW Spain) is considered one of the most important European wetlands, with many ecosystems depending on groundwater. As a consequence of intense groundwater withdrawals for urban use and, above all, irrigation, serious impacts have been observed in the eastern part of this aquifer, where the Doñana National Park is located. There is also groundwater exploitation in the western part of the aquifer, where a groundwater divide exists. The main goal of this work is to analyze the impact of groundwater withdrawals in this area. For this, the evolution of groundwater levels since 1968 in piezometers and pumping wells has been compiled and analyzed. This zone is characterized by the existence of a deep aquifer of high transmissivity, and a shallow aquifer with lower hydraulic conductivity, which behaves as an aquitard. Results show that pumping has caused a strong cone of depression in the deep aquifer, shifting the groundwater divide, and diverting water originally directed to the protected area, aggravating the overexploitation problems. Drawdowns in the shallow aquifer are lower and seem to be masked by the slow groundwater dynamic. However, a small lowering of the water table may cause severe impacts on such fragile ecosystems. The division of the aquifer for its management into six groundwater bodies belonging to two hydrographic districts has caused these important changes to go unnoticed until now. A proper coordination between the different water authorities managing groundwater and urgent adoption of remediation measures is essential.

## Introduction

**Fig. 1:**
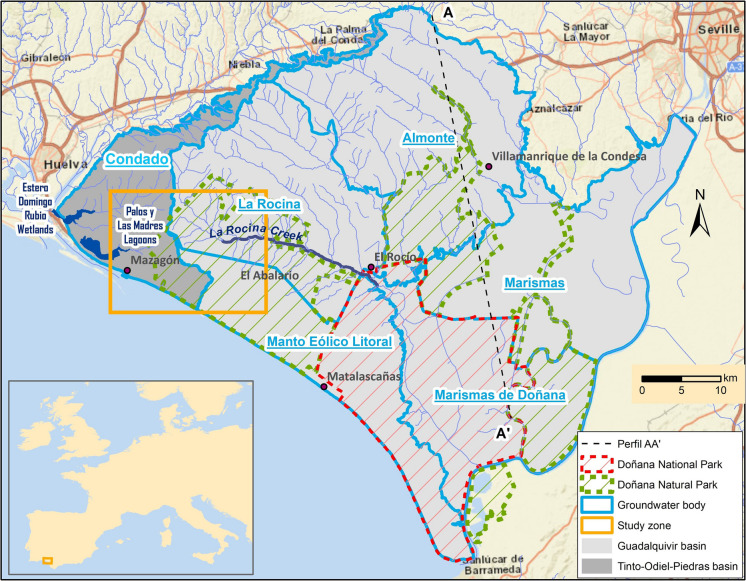
Map showing the Almonte-Marismas aquifer, the limits of the Doñana protected areas, and the 6 groundwater bodies into which the aquifer is divided: Condado, Almonte, La Rocina, Manto Eólico Litoral, Marismas, and Marismas de Doñana. The profile A-A’ is shown in Fig. 2, and the study zone is represented in Fig. 3

The Doñana Natural Space (Fig. 1) has a total surface of 1225 km^2^ and includes the Doñana National Park, created in 1969, and the Doñana Natural Park, established afterwards with a lower level of protection. Its extensive surface and situation, just between Europe and Africa in the convergence of the Mediterranean and Atlantic regions, make this natural area one of the most important wetlands in Europe, being a strategic point within the global migratory bird networks (e.g., Camacho et al., 2022; Navedo et al., 2022). Doñana has been recognized as Biosphere Reserve and World Heritage Site by UNESCO and is also included as wetland of international importance within the RAMSAR Convention. Wetlands like Doñana provide important ecosystem services; however, most of these ecosystems have been destroyed or are strongly degraded (Green et al., 2017; Xu et al., 2024).

**Fig. 2:**
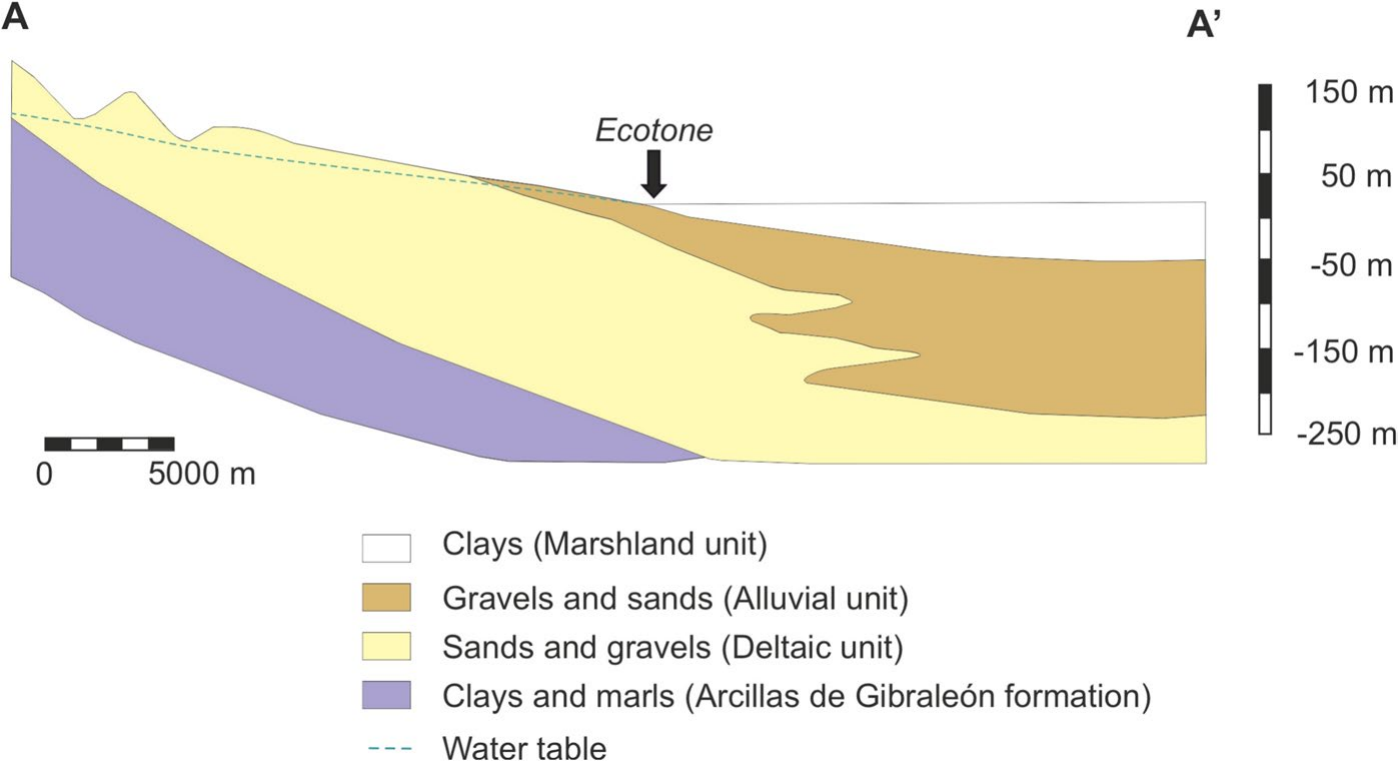
Hydrogeological cross section of the Almonte-Marismas aquifer along the line A-A’ indicated in Fig. 1 (adapted from Custodio et al., 2009)

**Fig. 3:**
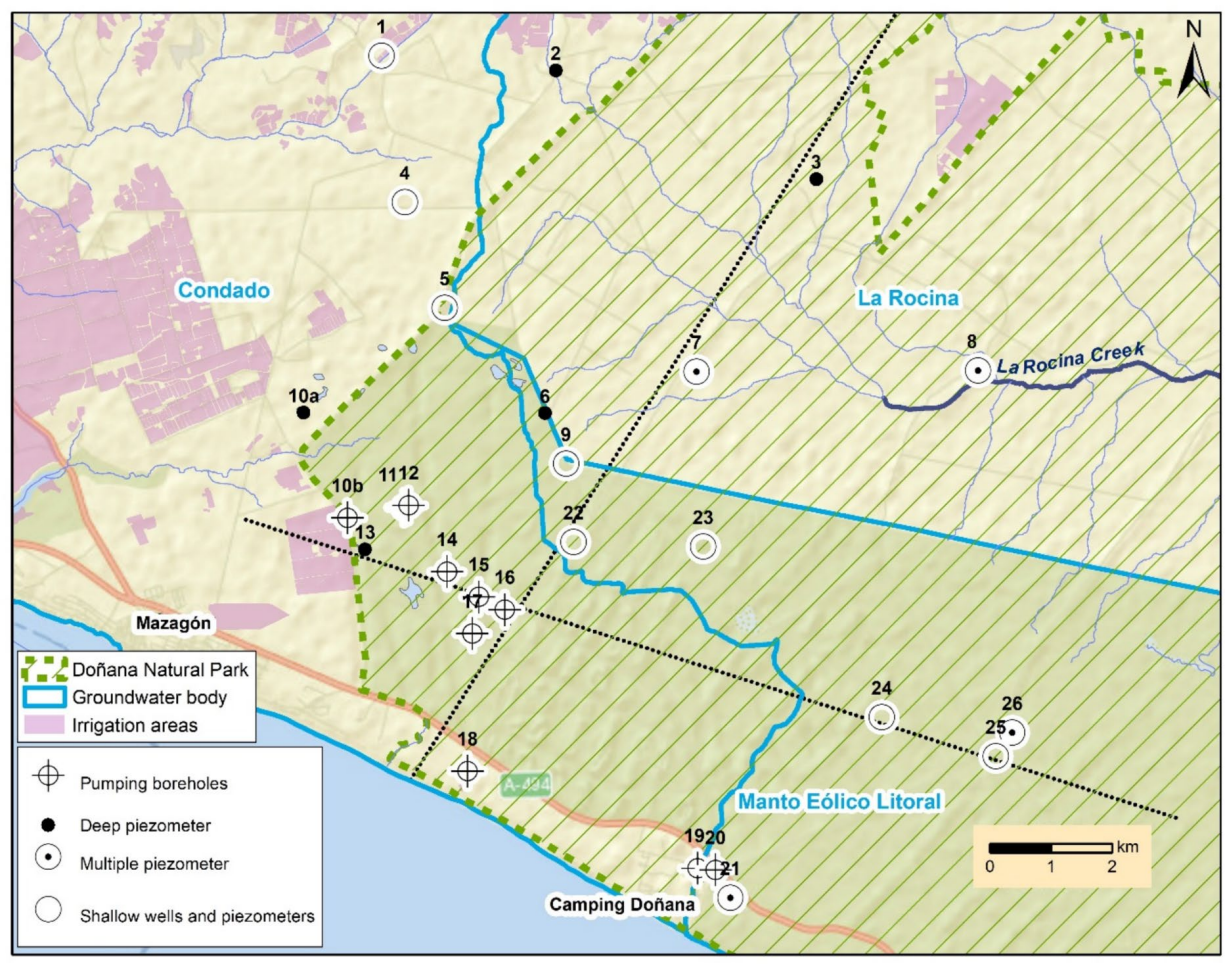
Study area within the southwestern part of the aquifer (El Abalario zone) including the groundwater monitoring point and the limits of groundwater bodies. The black dashed lines indicate the profiles shown in Figs. 8 and 9

Located in a flat area, Doñana presents two main environments clearly identified:

(i) The marshlands, which depend strongly on rainfall and runoff from its drainage watershed, being commonly flooded during winter and completely dried in summer (80% of the original surface of the marsh was transformed into agricultural areas, so that only 20% remains currently; Green et al., 2017).
(ii) A sandy area surrounding the marshlands with numerous lagoons considered as priority habitats by the European Union (up to 3000 lagoons have been identified within the National Park during rainy years; Díaz-Paniagua et al., 2010).

Doñana is located over an aquifer, known as Almonte-Marismas, with a surface of 2600 km^2^, which is noticeable greater than the protected area (Fig. 1). Different types of wetlands are identified in the sandy area, from ephemeral lagoons mainly fed by surface waters, which remains flooded just a few weeks after intense rainfalls, to permanent lagoons due to the constant supply of groundwater. This leads to the occurrence of different environments which notably contributes to the rich biodiversity found in Doñana (Diaz-Paniagua et al., 2010). Furthermore, groundwaters maintain a dense riparian forest in La Rocina stream (Fig. 1), as well as vast areas vegetated with phreatophyte shrubs where the water table is close to the surface (commonly < 1 m). In addition, a transition ecotone is observed in those areas where marshlands and sandy areas are put into contact (Fig. 2), characterized by having a substrate with permanent moisture due to groundwater discharges (Acreman et al., 2022; Camacho et al., 2022; Custodio, 2010; Custodio et al., 2009; Green et al., 2024).

The exploitation of the aquifer started in the 1970s, promoted by the Food and Agriculture Organization of the United Nations (FAO) and the Spanish National Government, to improve the economic development in the area through irrigated agriculture. Simultaneously, numerous wells were drilled, mainly for strawberry cultivation, in private farms without any planning measures, many of them illegally. After years of unplanned development of agriculture in the area, the Regional Government of Andalusia legalized in 2014 around 9300 ha of irregular farms. However, there currently exists numerous illegal groundwater withdrawals (Green et al., 2024; Guardiola-Albert et al., 2024). In addition, there exist groundwater withdrawals for urban supply (although of less importance than for irrigation), mainly for the touristic towns of Matalascañas (Fig. 1). According to the hydrological official plans, the total volume of groundwater withdrawal is around 106 hm^3^, although some uncertainty around this value exists (Custodio et al., 2009). Thus, in 2021, the Court of Justice of the European Union condemned Spain for not considering within the hydrological plans illegal withdrawals for irrigation and those devoted to urban use. The Court also considered a lack of actions to avoid the alteration of Doñana´s habitats as a consequence of groundwater withdrawals (sentence C-559/19 of 24th June 2021; Green et al., 2024).

The European Union Water Framework Directive (WFD) allows aquifers to be split into different zones, so the Almonte-Marismas aquifer was divided into six groundwater bodies (Fig. 1), five of them (“Almonte,” “La Rocina,” “Manto Eólico Litoral,” “Marismas,” and “Marismas de Doñana”) belonging to the Guadalquivir River Basin District, which reports to the National Government, and the remaining (“Condado”) belonging to Tinto-Odiel-Piedras River Basin District, under the Andalusian Regional Government. Some of the boundaries between the groundwater bodies have a hydrogeological reason, for example, the limit between the masses “Manto Eólico Litoral” and “Marismas de Doñana” corresponds to the contact between the unconfined and confined aquifer (see the Study Zone section). Other limits are due to administrative or management reasons. For example, the boundary between “Manto Eólico Litoral” and “La Rocina” corresponds to the old delimitation of the Doñana Natural Park. This limit does not make any sense today because in this zone the Natural Park was expanded to the North in 2016, as can be seen in Fig. 1. In 2020, the Guadalquivir River Basin Water Authority (Confederación Hidrográfica del Guadalquivir, hereon CHG) declared three groundwater bodies (La Rocina, Almonte and Marismas; Fig. 1) at risk of failing to achieve the “good status” under the WFD due to the lowering of piezometric levels of up to 20 m within the areas of higher agricultural activities (Fig. 1). This has led not only to a decrease of groundwater inputs to the streams in this area, especially towards La Rocina stream (Fig. 1) and the ecotone but also the loss of wetlands, the decrease of direct evapotranspiration from the aquifer, and consequently, changes in vegetation with shift to xerophytic populations (e.g., Llamas, 1988; Suso & Llamas, 1993; Muñoz-Reinoso, 2001; Custodio et al., 2009; Muñoz-Reinoso et al., 2020; Green et al., 2024). Withdrawals for urban use in the surroundings of Matalascañas (Fig. 1) have also affected the lagoons of the National Park area, in such a way that the closer ones to the pumping wells have totally dried, while in the farther ones, the hydroperiod is decreasing (e.g., Serrano & Serrano, 1996; Manzano et al., 2005; Díaz-Paniagua & Aragonés, 2015; Fernández Ayuso et al., 2018; Rodríguez-Rodríguez et al., 2021; Acreman et al., 2022; de Felipe et al., 2023). Finally, there are pollution problems in groundwaters associated to the use of fertilizers in agriculture (e.g., Olías et al., 2008; Custodio et al., 2009: Kohfahl et al., 2019).

All these previous works focus on the eastern part of the aquifer, in the Doñana National Park area, while information on the impacts of groundwater withdrawals in the southwestern area is limited (Trick, 1998; Trick & Custodio, 2004). In this area, a groundwater divide is found, limiting the “Condado” groundwater body, belonging to the Tinto, Odiel and Piedras River Basin District, from “Manto Eólico Litoral,” “La Rocina,” and “Almonte” bodies of the Guadalquivir District (Fig. 1). While the groundwater flows to the west in the “Condado” groundwater body, the opposite flow direction is observed in the Guadalquivir bodies, with groundwater directed towards the Doñana National Park. Close to the water divide, groundwater withdrawals for irrigation are taking place in the “Condado” groundwater body. In addition, important pumping (1 hm^3^/year) occurred for urban use in Mazagón until 2016. In this context, the main objective of this work is to analyze the impact of extractions in the “Condado” groundwater body on the aquifer water contributions to the wetlands of Doñana Natural Space.

## Study area

From a geological point of view, the Almonte-Marismas aquifer is composed of sedimentary materials from the Guadalquivir basin, mainly Neogene and Quaternary formations. The sequence, from bottom to top, starts with materials from the “Arcillas de Gibraleón” Formation, which have a deep marine origin and are constituted by clays and marls from the final phase of Miocene and beginning of Pliocene (Salvany et al., 2010). Over these marls, four different Plioquaternary units are deposited (Fig. 1):

- Deltaic unit, constituted mainly by sands and silts.
- Aeolian unit, developed principally over the coastal strip and formed by very homogeneous fine sands.
- Alluvial unit, formed by gravels and sands, which are mainly found below the current marshlands of the National Park.
- Marshland unit, constituted chiefly by clays and deposited over the alluvial unit (Salvany et al., 2010).

The study area is located in the western part of the aeolian unit, known as El Abalario, which is part of the Doñana Natural Park (Figs. 1 and 3). In this zone, the highest altitudes of Doñana are found (maximum of 106 m), which forms a sharp cliff in the coastal area due to marine erosion. The main geological units in this area are (Salvany et al., 2010) (1) Almonte Sands and Gravels Formation of fluvial origin and age of Late Plioceno-Early Pleistocene, composed by coarse sands and gravels which forms a continuous layer, with a thickness between 15 and 26 m, below the aeolian sands in the Abalario zone; (2) Abalario Sands Formation, constituted by medium and fine aeolian sands (some dunes are still active; Goy et al., 2022), deposited over the previous formation. It forms a sedimentary body of Quaternary age with a maximum width of 150 m to the east of the study area, decreasing progressively to the north and west. Some organic-rich layers with ferruginous crusts and clay levels can be locally identified. There exist numerous temporal small lagoons associated to interdune low areas (Custodio et al., 2009; Manzano et al., 2013), with different hydrological conditions depending on its relationship with the aquifer.

The climate in Doñana is of Mediterranean type, with mild and rainy winters and hot and dry summers. The average annual temperature is around 18–19 °C, with minimum temperatures rarely below 0 °C and maximum values sometimes above 40 °C in summer (Custodio et al., 2009). The average rainfall in the area is around 550 mm, mainly collected from October to March, and a large dry period from June to September. However, rainfall varies widely through the years, with extreme values ranging from less than 200 mm to above 1000 mm (CHG, 2022). Nonetheless, it is expected that annual precipitation will decrease in the next years but rainy events will be more intense because of climate change (Guardiola & Jackson, 2011).

No people live in El Abalario area, except in the touristic towns located on the coast (Matalascañas and Mazagón; Fig. 1). This area was extensively reforested with eucalyptus in the middle of the twentieth century (Sousa Martín & García Murillo, 1999). However, most of them were replaced by autochthonous plants in the 1990s, mainly shrubs, which led to the recovery of the water table (Trick & Custodio, 2004). In addition, intense agricultural activities by irrigation have been developed to the north and especially the west of the study area. Although most waters used come from surface sources, some farms still use groundwater.

Regarding the hydrogeological behavior of Almonte-Marismas aquifer materials, the “Arcillas de Gibraleón” clays and marls have a low permeability and constitute the impermeable base of the system, while those materials from the deltaic, alluvial, and aeolian units are permeable (Custodio et al., 2009). Finally, clays from the Doñana marshland unit also have an impermeable behavior. As a consequence, the aquifer can be separated into two different parts:

1. The zone where sandy materials outcrop, constituting an unconfined aquifer which is fed directly by infiltration waters from rainfall.
2. The current Doñana marshlands zone, where the aquifer is confined by the outcropping clay-rich sediments.

The main source of recharge is the direct infiltration of rainfall over the unconfined part, although a secondary source is the return water from irrigation. It is estimated that the recharge by rainfalls in El Abalario zone is between 100 and 200 mm/year (Trick & Custodio, 2004). The water table is generally a few meters below the surface. In parallel to the coast, there exist a dome in the water table of El Abalario area. In the western zone (the Condado groundwater body), the groundwater flows towards the Tinto River, in the coastal zone flows into the sea and in the rest of the area the groundwater flows towards the La Rocina Creek or the National Park zone. In this context, the natural discharge of the aquifer takes place by (i) discharge into the different streams draining the area, (ii) discharge into the sea in the coastal zone, (iii) discharge through the ecotone (contact between sandy materials and Doñana marshland), and (iv) through evapotranspiration by vegetation in those zones where the water table is close to the terrain surface. In addition, as commented before, important outflows take place since 1970 because of groundwater extractions.

The impermeable clays and marls sink progressively to the southeast; therefore, the thickness of the aquifer increases in such direction. The alluvial materials exhibit the highest permeability and reach their maximum development below the Doñana marshlands, so the highest transmissivity of the aquifer (values above 1000 m^2^/day) and the most productive wells (flows > 100 L/s) are found in this sector (Custodio et al., 2009). In El Abalario area, the transmissivity decreases to the west, with values below 100 m^2^/day in the Mazagón sector (Trick & Custodio, 2004). The storage coefficients in this area are very low (between 10^−3^ and 10^−4^) due to the higher transmissivity of the Almonte Sands and Gravels Formation, located in depth, in relation to the medium and fine sands of Abalario Formation. Consequently, in this zone, the aquifer really behaves as semiconfined (Trick & Custodio, 2004).

## Methodology

The methodology followed in this work is based mainly on the compilation of hydrogeological information and historical data of groundwater level obtained from monitoring networks of different organisms (Geological Survey of Spain, Water Authority of the Andalusian Regional Government, Guadalquivir Hydrographic Confederation and Doñana Natural Space). Although there are some data on piezometric levels since the end of 1960s (Table 1), most piezometers were installed from 1990 (some of them multilevel), from where there are regular data (Kohfahl et al., 2019). The validity of data has been checked after graphical representation of the entire dataset, removing those anomalous values (i.e., measurements showing a sharp variation before recovering immediately its former value). In addition, a field campaign was performed to measure the groundwater monitoring points. Despite the probable existence of more pumping wells for irrigation to the west of this area, there is not available data. From piezometric and lithological information collected in the monitoring points, two hydrogeological cross sections along the study area in natural and current conditions were drawn (flow lines were drawn manually taken into account that groundwater moves mainly vertically in the shallow aquifer and horizontally in the deep aquifer).

**Table 1:** Main characteristics of groundwater monitoring points

No	Type	Name/code	UTM coordinate (zone 29N)		Altitude (m)	Depth (m)	Data series
			*X*	*Y*			
1	Shallow well	104,150,071	696,774	4,121,640	46.7	7.1	1989–2019, some gaps
2	Deep piezometer	05.51.057	699,633	4,121,396	55.9	31	Since 2009
3	Deep piezometer	104,160,019	703,900	4,119,620	58	30.5	Since 1989
4	Shallow well	04.14.006	697,154	4,119,240	49	14	Since 2000, some gaps
5	Shallow well	104,150,004	697,811	4,117,515	62.4	10.8	One data in 1966 and since 1993
6	Deep piezometer	104,160,022	699,454	4,115,790	58	55	Since 1994
7	Multilevel piezometer	La Matilla	701,937	4,116,471	48	24	Since 2007
						89	
8	Multilevel piezometer	Bodegones	706,550	4,116,490	32	9	Since 1994
						58	
9	Shallow well	104,220,016	699,801	4,114,968	64.1	4.7	1991–94 and since 2006
10a	Deep borehole	104,150,013	695,500	4,115,800	53	64	Sporadic dada
10b	Pumping borehole	-	696,216	4,114,078	56	-	No
11	Pumping borehole	104,210,062	697,145	4,114,252	57	-	Sporadic data
12	Pumping borehole	104,210,063	697,217	4,114,282	60	-	Sporadic data
13	Deep piezometer	104,210,004	696,504	4,113,564	57.2	28	Since 1968
14	Pumping borehole	104,210,065	697,850	4,113,200	60	92	Sporadic data
15	Pumping borehole	104,220,010	698,369	4,112,790	65	86	Sporadic data
16	Pumping borehole	104,220,009	698,794	4,112,580	64	86	Sporadic data
17	Pumping borehole	104,220,011	698,264	4,112,190	62	88	Sporadic data
18	Pumping borehole	104,220,008	698,184	4,109,940	44	90	Sporadic data
19	Pumping borehole	1988/00280	701,952	4,108,350	45	-	No
20	Pumping borehole	1988/00280	702,243	4,108,320	46	-	No
21	Multilevel piezometer	Arenosillo	702,492	4,107,866	38	22	Since 1993
			702,494	4,107,868	39	60	
			702,499	4,107,868	39	98	
22	Shallow piezometer	Laguna Moguer	699,920	4,113,683	66	12.4	1993–97 and since 2001
23	Shallow piezometer	Laguna Norte Vaca	702,045	4,113,608	65	12.3	1993–97 and since 2001
24	Shallow piezometer	Laguna Río Loro	704,971	4,110,818	67	12.3	1993–97 and since 2001
25	Shallow well	El Abalario	706,839	4,110,181	66.6	7.6	Since 1968
26	Multilevel piezometer	El Abalario	707,110	4,110,570	65	25	Since 1994
						72	
						108	

Monthly rainfall data for the period 1970–2021 were obtained from CHG (2022). Data from 1965 to 1969 has been completed from a regression curve determined with data from the meteorological station Huelva Este, belonging to the Spanish Meteorological Agency, which is located 18 km away from the study area. From monthly precipitation data, the cumulative deviation from mean (CDM) has been obtained in order to identify periods especially dry and rainy. First, the mean monthly precipitation from the data series was obtained (*P*_mean_). Then, the deviation (*D*_*i*_) was calculated for each month by subtracting *P* from each observation in the period (*D*_*i*_ = *P*_*i*_ − *P*_mean_). These values are then summed in sequence (Smail et al., 2019):

$${\text{CDM}}_{i}={\sum }_{j=1}^{i}{D}_{j}$$

## Results and discussion

### Historical evolution of groundwater levels

#### Rainfall evolution

**Fig. 4:**
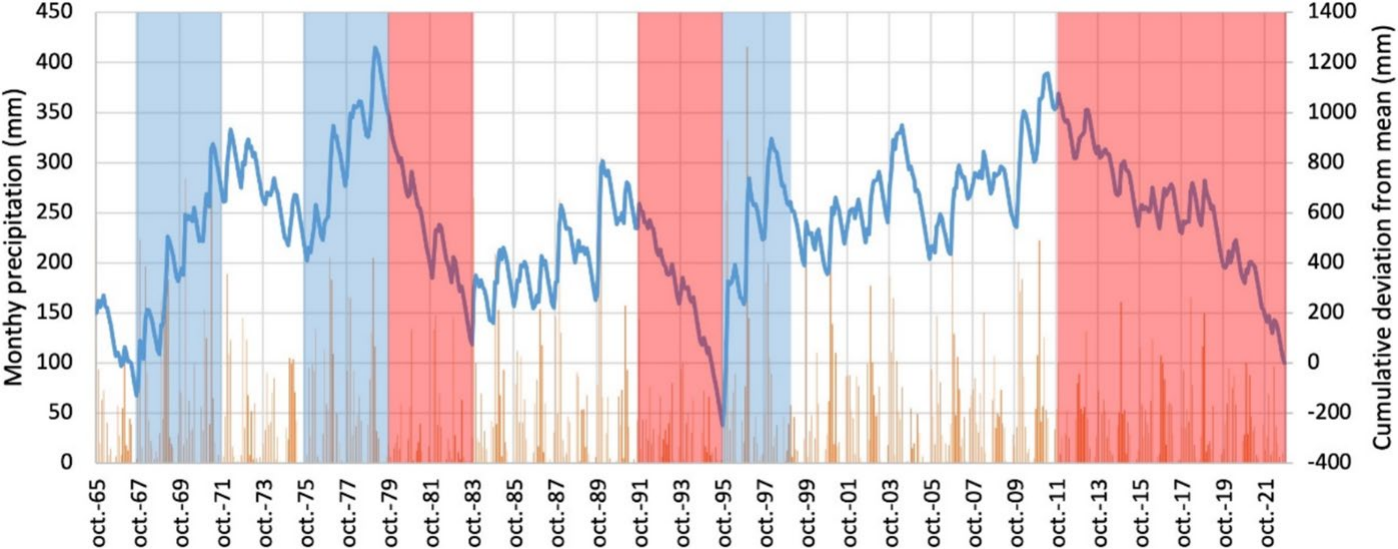
Monthly precipitation (orange bars) and cumulative deviation from its mean (blue line). Wet periods are indicated by blue shaded areas and dry periods by red ones

The average annual precipitation during the period 1965 to 2022 was 534 mm. Nonetheless, there was a great variation range, with minimum values of 176 mm in 2004/05 and maximum of 1000 mm in 1995/96. The CDM evolution (Fig. 4) indicates the existence of three main rainy periods:

1. From 1967/68 to 1970/71 (average rainfall of 748 mm).
2. From 1975/76 to 1978/79 (average of 665 mm).
3. From 1995/96 to 1997/98 (average of 840 mm) (Fig. 4).

Conversely, the main dry periods were (1) from 1979/80 to 1982/83 (average rainfall of 305 mm), (2) from 1991/92 to 1994/95 (average of 348 mm) at the end of which the lowest CDM value of the study period is observed, and (3) from 2011/12 to 2021/22 (average of 422 mm), a less pronounced but longer dry period.

#### Evolution of groundwater monitoring points located to the east

**Fig. 5:**
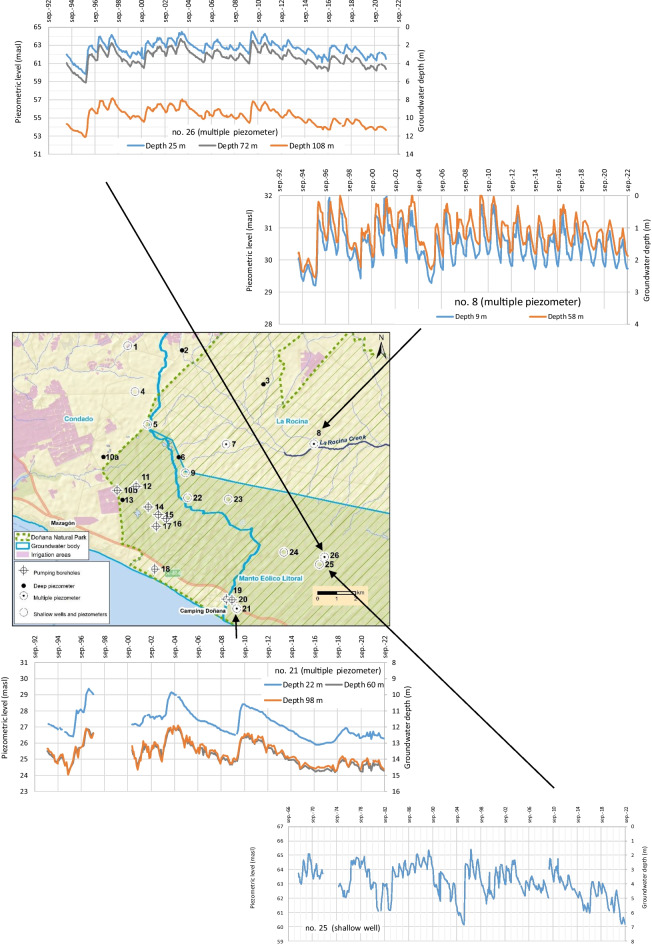
Evolution of piezometric level and groundwater depth at some monitoring points located to the east, away from main abstraction areas (masl: meters above sea level). Red arrows inside the graphs indicate the minimum value of CDM

The evolution of groundwater levels in those points with more complete dataset is shown in Fig. 5. The monitoring points to the east of the study area are located far away from pumping sites (Fig. 3). Monitoring point no. 25 is a shallow well with a long dataset starting in 1968 (Fig. 5). In natural conditions, CDMs should correlate well to observed groundwater level change (Naranjo-Fernández et al., 2020; Smail et al., 2019). It can be seen that the groundwater level range from 2 m during the rainy periods to 7 m during the driest, coinciding with the minimum value of CDM (year 1994/95; Fig. 4). A tendency to decrease is observed since 2011 due to the reduction of rainfalls. However, the groundwater level in 2022 is slightly higher than in 1995.

In the multilevel piezometer no. 26, located near the previous one, data have been registered since 1994 (Fig. 5). The minimum value was reached in 1995, coinciding with the lowest value of CDM. In 2022 the groundwater level is clearly higher than in 1995. On the other hand, these points are located in the recharge zone of the aquifer, as evidenced by the downward vertical gradient (the groundwater depth is lower as the piezometer depth increases; Fig. 5). The strong hydraulic gradient between the piezometers placed at 72 and 108 m depth is related to the existence of a clay-rich layer with lower permeability in this area (Trick, 1998).

Although less pronounced, this downward vertical gradient is also observed in the multilevel piezometer no. 21 (Fig. 5) located to the south, close to the coast. The deepest piezometers (reaching 60 and 98 m depth) exhibit a similar evolution with fluctuations associated to groundwater withdrawals to supply a camp site during summer. This pumping (250.000 m^3^/year) has not caused a significant decrease in groundwater levels (values in 2022 are similar or even slightly higher than those recorded in 1995).

The multilevel piezometer no. 8, located to the north, next to La Rocina stream, is on the contrary located in the discharge zone of the aquifer (Fig. 3). Thus, it shows an upward vertical gradient (the piezometric level in the deep piezometer is higher than that in the shallower one). During the rainy years the water depth in the deep piezometer is zero (water at the surface), behaving as a flowing well. The minimum value was reached in 1995 while clearly higher values are recorded in 2022, coinciding with CDM evolution (Fig. 3). The replacement of eucalyptus populations by autochthonous shrubs in the 1990s could have helped to increase the groundwater levels in this sector.

#### Evolution of groundwater monitoring points located close to pumping sites

**Fig. 6:**
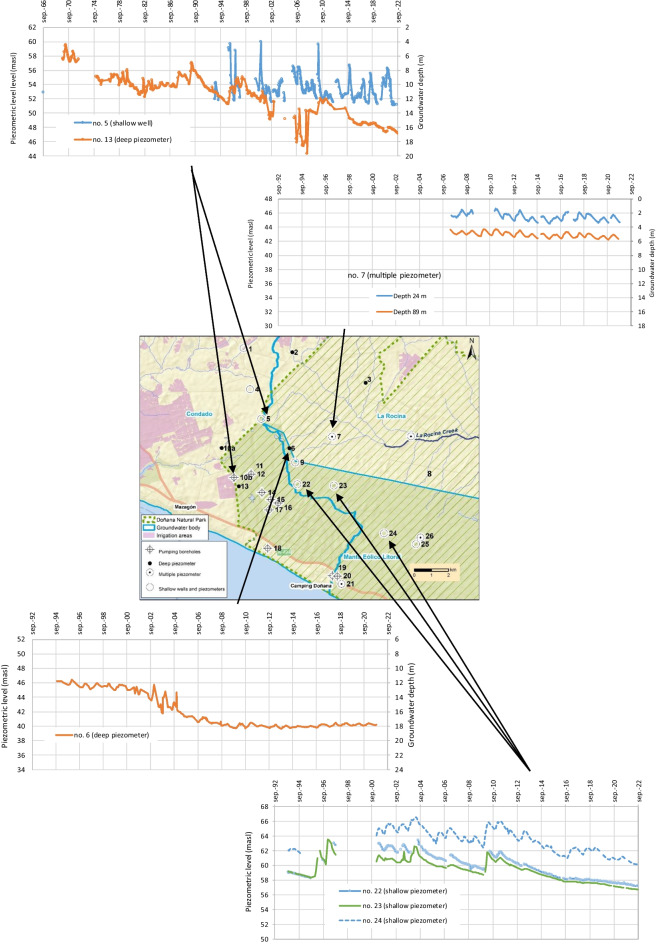
Evolution of piezometric level and groundwater depth at some monitoring points located near the main abstraction zone (masl: meters above sea level)

The dataset of piezometer no. 13, located close to the pumping at NE of Mazagón, starts in 1968 and displays a clear and prolonged decreasing tendency (Fig. 6). The groundwater level has decreased more than 10 m since 1970, showing a direct influence of pumping. While the groundwater withdrawals for urban supply of Mazagón stopped in 2016, no recovery of level has yet been observed. In the shallow well no. 5, located 4 km north of piezometer no. 13, dataset starts regularly in 1993 but no clear tendency is observed (this will be explained below). Nonetheless, a decrease in water depth of around 6 m is observed from 2000 to 2008 in a deeper piezometer (no. 6; Fig. 6), approximately to the same distance with respect to pumping sites. From 2008 onwards, the groundwater level seems to have been stabilized. This point is on the Guadalquivir district (between groundwater bodies La Rocina and Manto Eólico Litoral), while the pumping locations are in Condado groundwater body. That is to say, this monitoring point is located beyond the groundwater divide which naturally separate the zone where groundwater flows to the west and to the east. The piezometric decreasing beginning in 2000 (Fig. 6) indicating the groundwater divide was being moving towards the northeastern because of the pumping, reaching this monitoring point in that year. The stabilization of groundwater level approximately from the year 2008 was probably caused by the enlargement of the capture zone of the pumping.

The multilevel piezometer no. 7 (Fig. 6), located northeast of piezometer 6, has data only since 2007. A decreasing tendency is observed because rainfall in the last 12 years has been below average, but both the shallow and deep piezometers have the same evolution, so they do not seem to be affected by the pumping in the Mazagón area.

On the other hand, monitoring points nos. 22, 23, and 24 correspond to shallow piezometers (12 m depth) drilled in 1993. Although some data are missing (Fig. 6), it is striking that piezometers no. 22, no. 23, and the shallow well no. 5, located close to pumping sites, do not record the sharp decreases observed in other monitoring points (i.e., 6 m in piezometer nos. 6 and 11 m in piezometer no. 13). This is explained by the existence of two different aquifer levels:

1. A deep aquifer constituted by sands and gravels, with higher transmissivity, which feeds the withdrawal wells.
2. A shallower aquifer level formed by aeolian medium and fine sands, with a lower hydraulic conductivity, which behaves as an aquitard (Trick & Custodio, 2004).

As a consequence, the clear decreases in water level associated with pumping from the lower aquifer level are smoothly reflected in the water table. However, the groundwater depth at the end of the study period in piezometers nos. 22 and 23, closer to the pumping sites, was around 1 m lower than that recorded during the intense drought of 1995 (Fig. 6), which seems to indicate the influence of deep withdrawals on the water table. In this sense, this decrease is not observed in a shallow piezometer farther from the pumping sites (no. 24).

#### Evolution of groundwater monitoring points located to the north

**Fig. 7:**
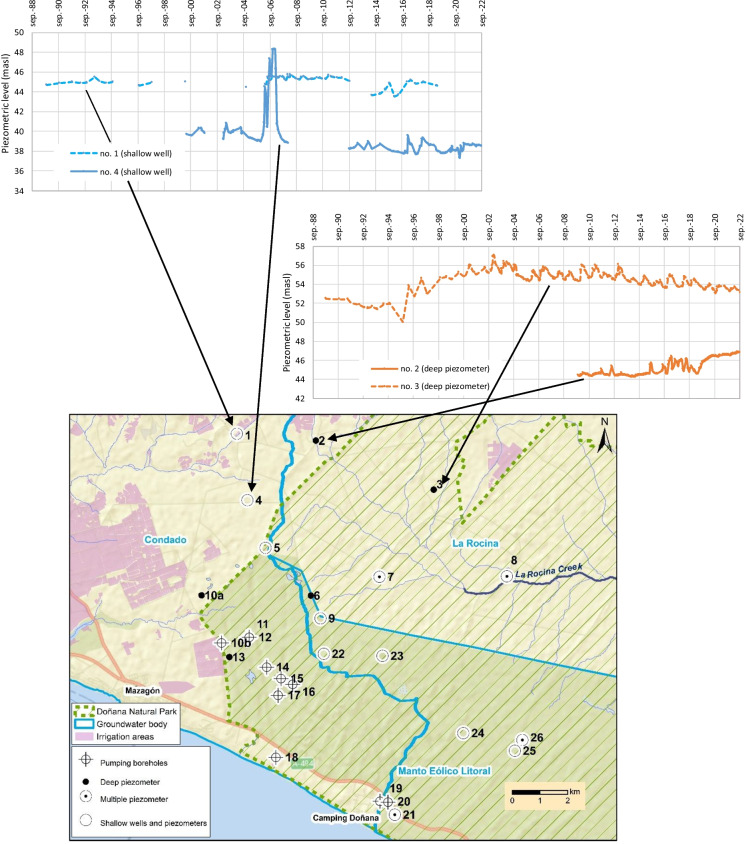
Evolution of piezometric level at some monitoring points located to the north (masl: meters above sea level)

The evolution of the water table in the monitoring wells located to the north is variable. In the shallow well no. 1, levels seem to have decreased since 2014, although there is no data since 2019 (Fig. 7). Piezometer no. 4 shows a period from 2005 to 2006 with anomalously high values. However, it appears to exist a decreasing tendency, with a drop of around 2 m from 2000 to 2022. An increasing tendency in water levels is observed in piezometers no. 2 (since 2019) and no. 3 (since 1995), as a result of the replacement of groundwater by surface water for irrigation in this sector (MITERD, 2022).

### Natural conditions and current situation

Declines in water level are much greater in the deepest monitoring wells than in the shallowest ones (Fig. 6). For instance, the decrease observed in the piezometer no. 6, located 2 km away from the pumping sites, is around 6 m, while that in piezometer no. 22, located closer to the pumping sites, is only 1 m. This fact has been also observed in other zones of the Almonte-Marismas aquifer and is attributed to the lower hydraulic conductivity of fine aeolian sands (with occasional intercalation of clay levels) with respect to the alluvial gravels and sands located in the aquifer bottom, which exhibit a high transmissivity (CHG, 2022; Green et al., 2024; Trick & Custodio, 2004). Thus, the aeolian sands behave as an aquitard, where water mainly flows vertically, while in the gravel and sands level the water flows horizontally and the decrease in water levels driven by pumping are noticeable at high distances (Green et al., 2024; Trick & Custodio, 2004).

**Fig. 8:**
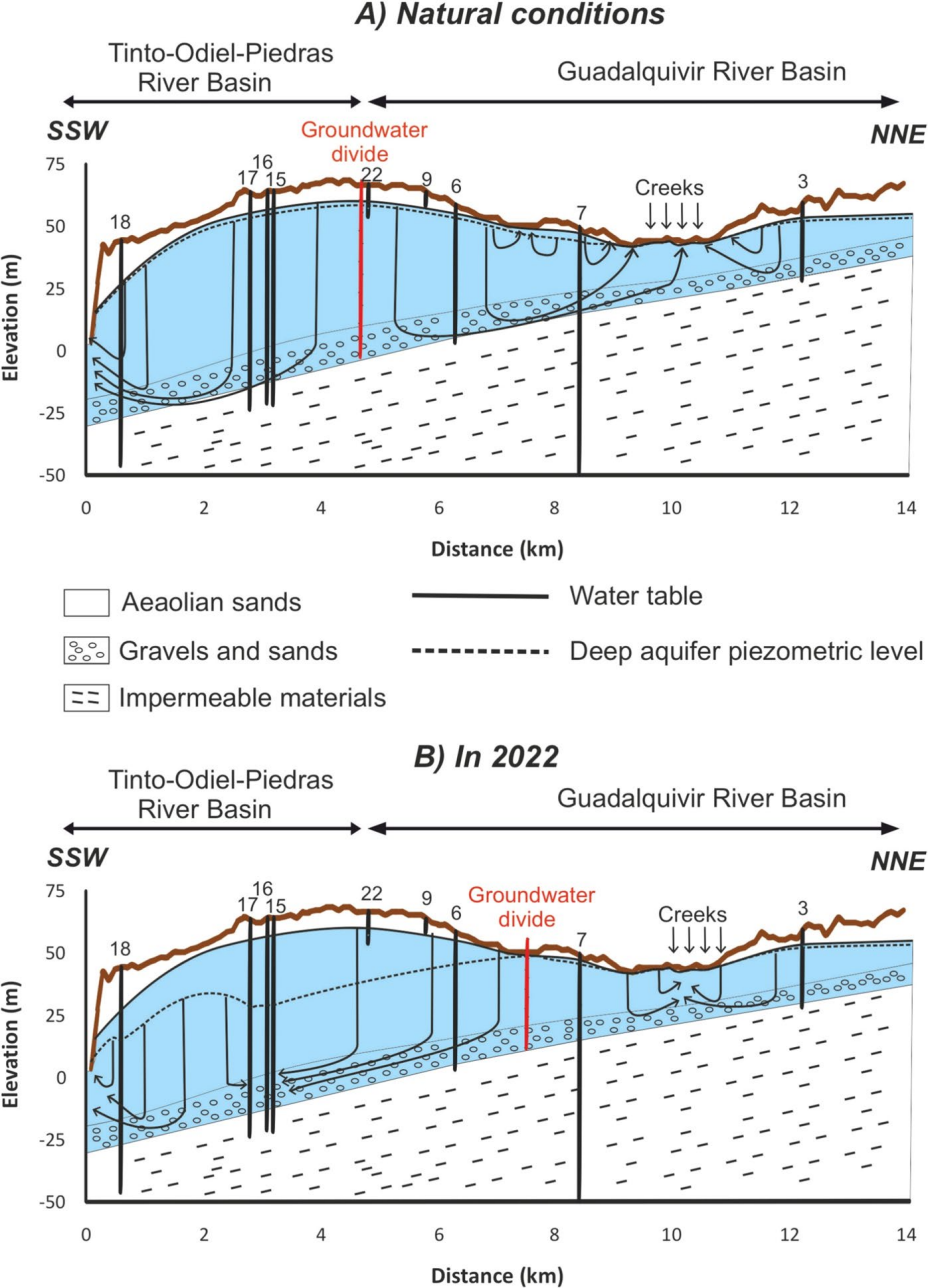
Schematic groundwater levels and flow in a SSW-NNE cross section in natural conditions and in 2022 (see Fig. 2 for location). The projection of wells and piezometers close to the profile is shown

**Fig. 9:**
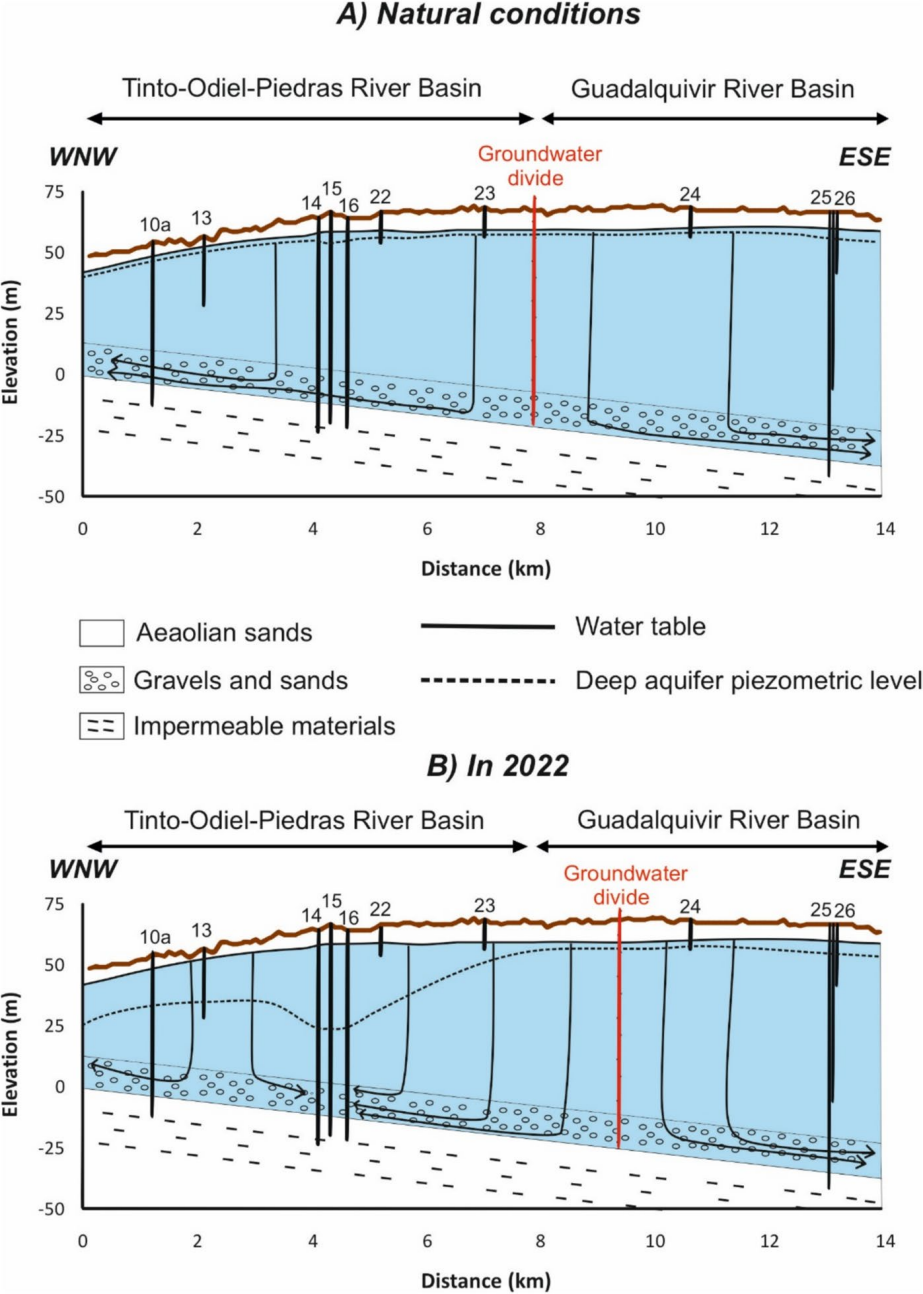
Schematic groundwater levels and flow in a WNW-ESE cross section in natural conditions and in 2022 (see Fig. 2 for location). The projection of wells and piezometers close to the profile is shown

The groundwater levels upon natural and current conditions has been graphically represented (Figs. 8 and 9). The location of the divide was determined approximately considering the piezometric levels of the deep aquifer. That is, the divide must be located between the two monitoring point with the highest piezometric level. In natural conditions, the piezometric level of the deep gravel and sands is slightly lower than the water table in the aeolian sands in the recharge zones but slightly higher in the discharge zones. However, both levels are quite similar, following the topography. Groundwater flows vertically through the upper aeolian sands and horizontally through the gravels and sands, rising afterwards to the discharge zones in the coast or in La Rocina stream headwaters (Fig. 8). Local upwards flows through the aeolian sands are also identified, which feed some wetlands as previously reported in a mathematical model by Trick and Custodio (2004).

Due to the intense exploitation of the aquifer, especially at the southwestern sector, and the subsequent water level decreases, significant changes in the system have occurred. Importantly, the groundwater divide has been displaced around 4 km to the northeast, in such a way that groundwater within an important sector of La Rocina groundwater body is now flowing towards the Condado groundwater body (Fig. 8). The groundwater divide has also been displaced towards the east, altering the prevailing groundwater flows (Fig. 9). As a consequence, groundwater inputs towards La Rocina stream must have decreased. This fact may have remarkable ecological implications:

1. Changes in the phreatophytic populations in the discharge zone such as La Rocina stream, lagoons, and those areas where the water table is close to the surface. Although the decrease in water level observed in the aeolian sands is relatively small (< 1 m), it may have important ecological implications (Antunes et al., 2018; Custodio, 2010). A replacement of vegetal species by others with lower water requirements has already been identified to the west of this zone (Rodríguez-González et al., 2017) as a consequence of the important cone of depression located close to El Rocío town (Acreman et al., 2022; CHG, 2022; Green et al., 2024).
2. The decrease of water inputs from La Rocina stream to the marshes of the Doñana National Park. An important reduction of these inputs has been previously identified, also associated to the intense groundwater withdrawals in the surroundings of El Rocío (Manzano et al., 2013), although withdrawals from the surroundings of Mazagón may also play a role in such declines.
3. Apart from the impacts on ecosystems at the Doñana natural area, other wetlands located to the west of the study area (Palos and Las Madres Lagoons and Estero Domingo Rubio Wetlands, Fig. 1) may also be affected by the decrease in groundwater inputs driven by pumping. In this context, it is of paramount importance to evaluate the ecological impacts of the recorded groundwater level decreases.

Groundwater divides can shift because of natural causes, with significant environmental implications. Thus, Hajati et al. (2018) detected changes in groundwater-lake interactions due to the decrease of aquifer recharge, which affects the nitrate load received into the lake. Duque et al. (2011) showed the existence of seasonal groundwater divide shifts in a coastal aquifer linked to the infiltration of water from snowmelt along the river course, which controls groundwater quality. On the other hand, it is well known that groundwater divides can move due to pumping (e.g.De Smedt, 2014; Sheets et al., 2005). Our results show the complexity of this process with a two-layered aquifer system with a different response. It is noteworthy that the decreases of water level in the aeolian sands unit (behaving as an aquitard) are very slow and the resulting impacts may take longer to be detected. For example, using a numerical model, Custodio (2010) and Manzano et al. (2013) estimated that after any change caused in the aquifer, around 20 or 30 years would be needed to achieve intermediate conditions between the original (previous to the alteration) and the final state of the aquifer. In addition, the reductions in rainfall and the increases of temperatures associated to climate change will accentuate the decrease in the aquifer recharge rates, aggravating the groundwater level decline (Guardiola Albert & Jackson, 2011; Ramírez et al., 2018).

This important cone of depression due to the groundwater withdrawal in the surroundings of Mazagón has been neither detected by the Water Authorities of the Guadalquivir basin (belonging to the national government) nor that of the Tinto-Odiel-Piedras (belonging to the regional government). Consequently, the groundwater bodies of Condado and Manto Eólico Litoral are declared as bodies of good quantitative status according to the WFD, despite the severe affections by pumping shown in this work.

The compartmentalization of the aquifer into 6 different groundwater bodies and the apparent lack of coordination between both water authorities seems to have contributed to the unnoticed decreases in groundwater levels, despite being the aquifer with a more exhaustive monitoring network in Spain (Kohfahl et al., 2019). In addition, the limits between groundwater bodies are occasionally of mere administrative character, making difficult the quantification of groundwater fluxes among them (Guardiola et al., 2016).

The Spanish Government together with the Andalusian Regional Government has recently put into operation an ambitious plan to improve the aquifer situation and the ecosystems associated to Doñana (MITERD, 2022). Among the proposed measures are the closure of illegal wells, an exhaustive control of groundwater withdrawals, the replacement of groundwater by surface waters (from neighbors basins) for irrigation, the expropriation of lands with irrigation rights to decrease the withdrawals, financial aids to legal farmers to convert their crops from irrigated to rainfed and stop pumping water from the aquifer, and the formation of groundwater users associations. However, this plan is only focused in 5 out 6 groundwater bodies of the aquifer, thus ignoring the Condado groundwater body. To achieve a sustainable preservation of ecosystems in Doñana, it is essential to consider the aquifer as a whole, in place of the current compartmentalized structure, and to include remediation measures to recover the Mazagón sector.

## Conclusions

The Almonte-Marismas aquifer, upon which most Doñana ecosystems depend, is currently divided for management purposes into six groundwater bodies, dependent on two different hydrographic districts: Guadalquivir and Tinto-Odiel-Piedras. According to the WFD, coordination labors must be maintained between both authorities to guarantee an adequate management of the aquifer. However, this coordination has not been effective in the Almonte-Marismas aquifer, since important decreases in groundwater levels in the Mazagón area (close to the natural groundwater divide) have been unnoticed until now.

Groundwater with drawals in the area started around 1970 to satisfy irrigation and urban needs of Mazagón town. The decrease in groundwater levels driven by pumping in the deep aquifer is greater than 10 m and is observed at several kilometers. The pumping impacts on the shallow aquifer level are not so clear, with maximum decreases of the water table close to 1 m. Despite the low magnitude of these declines, they may have significant ecological implications, as some aquatic ecosystems and phreatophytes from the regional (i.e., La Rocina stream) and local (e.g., small lagoons in the area) discharge zones depend strongly on the shallow aquifer level. Although the groundwater withdrawals for urban supply of Mazagón stopped in 2016, the evolution of groundwater levels still has not recovered, which seems to indicate that these decreases may be associated to pumping for irrigation.

Finally, the groundwater withdrawals have caused a displacement of the groundwater divide of several km towards the east and northeast, leading to a change in groundwater regional fluxes, which main consequence is a decrease in groundwater inputs to La Rocina creek, one of the most important streams that provide water to Doñana marshlands. Due to the slow dynamic of groundwater the ecological impact could be currently masked and may take longer to be observed. Therefore, it is of paramount importance to investigate the ecological implications caused by such declines at La Rocina stream headwaters as well as at the lagoons located in the western zone of El Abalario. On the other hand, the described impact of the groundwater divide due to pumping in a two-layered aquifer with different transmissivity constitutes a case of interest to the international scientific community.

## Data Availability

Data sets generated during the current study are available from the corresponding author.
